# Subjective effects of the sleep position trainer on snoring outcomes in position-dependent non-apneic snorers

**DOI:** 10.1007/s00405-018-5036-y

**Published:** 2018-06-12

**Authors:** L. B. L. Benoist, A. M. E. H. Beelen, B. Torensma, N. de Vries

**Affiliations:** 1 Department of Otorhinolaryngology Head and Neck surgery, OLVG West, Jan Tooropstraat 164, 1061 AE Amsterdam, The Netherlands; 2 Department of Otorhinolaryngology and Head and Neck Surgery, Erasmus University Medical Center, Rotterdam, The Netherlands; 3 Department of Anesthesiology, Leiden University Medical Center, Leiden, The Netherlands; 4 Department of Oral Kinesiology, ACTA, Amsterdam, The Netherlands; 5 Department of Otolaryngology and Head and Neck Surgery, Antwerp University Hospital, Antwerp, Belgium

**Keywords:** Non-apneic snoring, Positional therapy, Primary snoring, Habitual snoring, Position-dependent snoring, Sleep position trainer

## Abstract

**Purpose:**

To evaluate the effect of a new-generation positional device, the sleep position trainer (SPT), in non-apneic position-dependent snorers.

**Methods:**

Non-apneic position-dependent snorers with an apnea–hypopnea index (AHI) < 5 events/h were included between February 2015 and September 2016. After inclusion, study subjects used the SPT at home for 6 weeks. The Snore Outcome Survey (SOS) was filled out by the subjects at baseline and after 6 weeks, and at the same time, the Spouse/Bed Partner Survey (SBPS) was filled out by their bed partners.

**Results:**

A total of 36 participants were included and 30 completed the study. SOS score improved significantly after 6 weeks from 35.0 ± 13.5 to 55.3 ± 18.6, *p* < 0.001. SBPS score also improved significantly after 6 weeks from 24.7 ± 16.0 versus 54.5 ± 25.2, *p* < 0.001. The severity of snoring assessed with a numeric visual analogue scale (VAS) by the bed partner decreased significantly from a median of 8.0 with an interquartile range (IQR) of [7.0–8.5] to 7.0 [3.8–8.0] after 6 weeks (*p* = 0.004).

**Conclusions:**

Results of this study indicate that positional therapy with the SPT improved several snoring-related outcome measures in non-apneic position-dependent snorers. The results of this non-controlled study demonstrate that this SPT could be considered as an alternative therapeutic option to improve sleep-related health status of snorers and their bed partners.

## Introduction

Snoring is the result of airflow passing through the upper airway, which in turn causes vibrations in the soft tissues. It is indicative of increased resistance in the upper airway and is often associated with obstructive sleep apnea (OSA) [1–4]. The prevalence of snoring in the general population is between 20 and 60%, depending on the definition, measurements, and population variables. Significant gender differences are observed with an higher prevalence in men than in women [1, 5–9]. Non-apneic snoring or primary snoring is defined as snoring with less than five apneic and/or hypopneic events per hour of sleep. Primary (self-reported) snoring could, just like OSA, be associated with excessive daytime sleepiness and negative sleep pattern behaviors [6, 10, 11]. Some research suggests that self-reported non-apneic snoring also has important clinical implications such as increased risk for cardiovascular disease [12, 13]. Besides these comorbidities affecting the primary snorer, snoring can also have negative impact on the sleep quality of the bed partner [14–17]. Habitual loud snoring may result in couples choosing to sleep apart or resort to using earplugs to counteract the sound [14]. These aspects can have a negative impact on the psychosocial aspects and the intimacy in a couple’s relationship, and even may trigger marital disharmony or result in divorce [1].

Sleeping position can influence the severity of snoring; however, few studies have looked at position dependency in non-apneic snorers. Nakano et al. found that snoring time and snoring intensity were lower in the lateral position than in the supine position in non-apneic snorers [18]. Choi et al. described that in non-apneic snorers, snoring decreased when a subject adopted a non-supine position [19]. A retrospective study performed by Benoist et al. looked at position dependency in non-apneic snorers seeking clinical care and found that 65.8% of this group is position dependent [20]. These results are in line with studies performed in OSA patients, which show position dependency to be inversely related to disease severity [21, 22]. Approximately 56% of patients with mild OSA is position dependent, defined as having at least twice as many events in supine position compared to the other sleeping positions, while in severe OSA position dependency only occurs in 6% [21, 23–25]. Hence, the proportion of position dependency may be highest in non-apneic snorers, followed by mild and moderate OSA, and lowest in severe OSA.

Positional therapy (PT) is one of the treatment options for positional OSA (POSA) patients. Van Maanen et al. showed that PT with a new positional device, the Sleep Position Trainer (SPT), effectively reduces disease severity in mild-to-moderate POSA [26, 27]. PT has also been compared to oral appliances, and short- and long-term results show similar efficacy in mild-to-moderate POSA patients [28, 29]. Since the majority of non-apneic snorers are position dependent, these subjects may potentially benefit from PT. The aim of this study was, therefore, to evaluate the effect of the SPT in position-dependent non-apneic snorers.

## Methods

### Study design

This study was conducted in the OLVG West hospital (Amsterdam, the Netherlands) after approval from the local ethical committee. Non-apneic snorers were recruited for this study. According to the ICSD-3 criteria, patients had no complaints of insomnia, excessive daytime sleepiness, or sleep disruption attributable to snoring; moreover, a diagnostic polysomnography (PSG) to rule out OSA was performed. After informed consent, subjects received the SPT, which they had to use during sleep for a period of 6 weeks. Study subjects and bed partners filled out questionnaires at baseline and after 6 weeks of using the SPT.

### Polysomnography

PSG was performed in the Sleep Laboratory of OLVG West using a digital PSG (Embla A10, Broomfield, CO, USA). During an overnight stay, various parameters were measured. To define the apnea–hypopnea index (AHI), airflow was measured using a sensor in the nasal cannula. Snoring was assessed using a nasal cannula and a piezo element sensor attached to the cricoid. These sensors detected snoring sounds lasting longer than 300–3000 ms. This resulted in an index of snoring events per hour, which could be differentiated between the various sleeping positions. Sleeping position was registered using a position sensor (Sleepsense, St Charles, IL, USA). This sensor was placed at the midline of the abdomen to discriminate between the different positions: supine, lateral right, lateral left, prone, and upright position. The recorded data were analyzed using special software (Somnologica™ studio) and manually edited. To exclude OSA patients, an apnea was defined as the cessation of nasal airflow of more than 90% for a period of 10 s or longer in the presence of respiratory efforts. In accordance with the prevailing definition from the American Academy of Sleep Medicine (AASM) at that time, a hypopnea was scored whenever there was a greater than 30% reduced oronasal airflow for at least 10 s, accompanied by ≥ 4% oxygen desaturation from pre-event baseline. Non-apneic snorers were defined as subjects with a snoring index > 1 and an AHI < 5/h sleep.

### Inclusion criteria

Subjects were included after undergoing a full-night PSG under suspicion of OSA in the period between February 2015 and September 2016 at the OLVG West Hospital, Amsterdam, the Netherlands. The inclusion criteria were as follows: adult subjects with a bed partner; AHI < 5 events per hour of sleep; supine sleeping position between 10 and 90% of total sleep time (TST); and the snoring was position-dependent according to PSG records. This latter was calculated using the snoring index, which is defined as the frequency of snoring: the number of snore events per hour of sleep. Supine-dependent snoring was defined as a supine snoring index higher than the total non-supine snoring index. The main exclusion criterion was previous therapy with the SPT.

### Primary outcomes

To measure the severity of snoring and the impact to patients’ quality of life, a validated subjective questionnaire was used: the Snore Outcome Survey (SOS) and the Spouse/Bed Partner Survey (SBPS) [30]. The SOS comprises eight questions on a 5-point Likert scale that evaluated the duration, frequency, severity, and consequences of problems associated with sleep-disordered breathing (SDB), and snoring in particular [Cronbach’s α = 0.672 (pre), 0.748 (post)]. To evaluate the impact of snoring on the bed partner, the SBPS was used. This questionnaire consists of three items on a 5-point Likert scale and assesses the effect of the snoring on the bed partner [Cronbach’s α = 0.868 (pre), 0.731 (post)]. The SOS and SBPS scores range from 0 (worst) to 100 (best) [30].

### Secondary outcomes

Secondary outcomes were the severity of snoring and satisfaction using the SPT. To evaluate the severity of snoring, a numeric visual analogue scale (VAS) containing a score between 1 (no snoring) and 10 (severe snoring) was filled out by the bed partners, at baseline and after 6 week of using the SPT. The study subjects were asked a dichotomous question (containing yes or no) about satisfaction using the SPT for a period of 6 weeks. Furthermore, according to the study by Lee et al. response profiles were determined; VAS ≤ 3 post-treatment was defined as ‘major response’ and post-treatment VAS ≤ 5 plus SOS ≥ 60 defined as ‘fine response’ [31].

### Intervention: the sleep position trainer

**Fig. 1:**
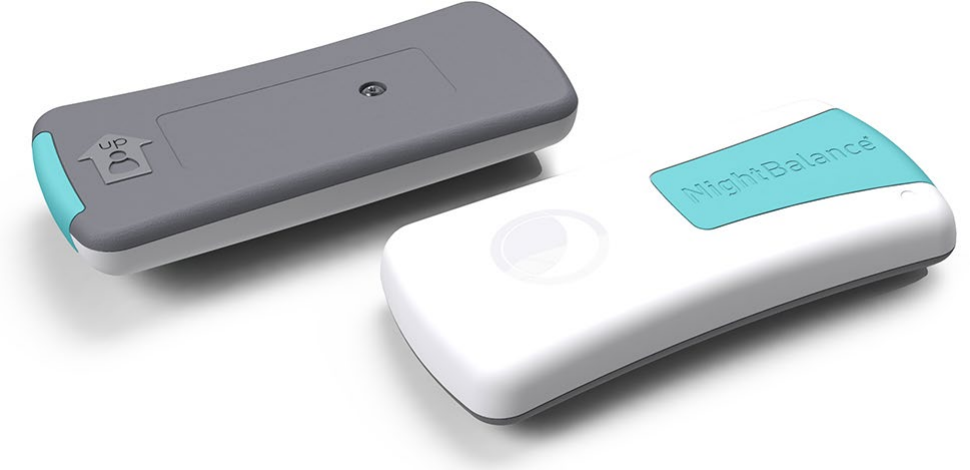
Sleep position trainer

**Fig. 2:**
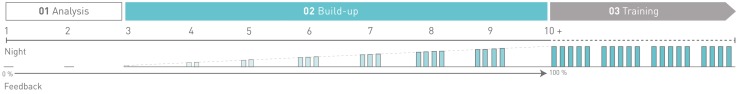
Various phases of the sleep position trainer. The analysis phase contains the first two nights in which no active feedback was given to the user. In the build-up phase, the next seven nights, the SPT started to vibrate in an increasing amount of episodes of supine position. During the training phase, night ten and onwards, the SPT vibrated every time a supine position was detected. If the subject did not react, the vibrations start again after a pause of 2 min

The SPT is a lightweight and small device (72 × 35 × 10 mm, 25 g), which is worn around the chest with a neoprene strap (Fig. 1). A three-dimensional digital accelerometer is used to determine body position. When lying in supine position, a subtle vibration is provided to give feedback to the user. The self-adaptive device gradually increases the intensity of the vibration until the user turns to a non-supine position. The vibration is adapted to the user in duration, strength, and pattern to maintain a timely response by the subject. Various phases are integrated for subjects to familiarize with the device: an analysis phase, a build-up phase, and a training phase (Fig. 2).

### Statistical analysis

For the analyses, different descriptive statistics and inferential statistics were used.

The SOS questionnaire test survey items use a Likert scaling model scoring system between 0 and 4. The sum score of the eight items, per individual score of the subject, could vary between 0 and 32. Total sum score was rescaled to an overall score between 0 and 100. If one question was left blank, scores were rescaled from 0 to 28 to overall scores between 0 and 100. However, when more than one item was left blank, SOS scores were treated as missing. The SBPS containing a Likert-scale model between 0 and 3 items that was rescaled to an overall score between 0 and 100 [30].

Categorical variables were expressed as n (%). Continuous normally distributed variables were presented by their mean and standard deviation and non-normally distributed data by their median and interquartile range for skewed distributions. Normally distributed continuous unpaired data were tested with the independent samples Student’s *t* test and in case of skewed data, with the independent samples Mann–Whitney *U* test. Normally distributed continuous paired data were tested with the dependent samples Student’s *t* test and in case of skewed data, with the Wilcoxon signed-rank test. Per-protocol analyses were completed for all outcome parameters. Significance level was set at *p* value of 0.05. Statistical analysis was performed using IBM SPSS statistical package 24.0.

## Results

A total of 36 subjects were included in the study. Six subjects (five males and one female) did not complete the follow-up and were excluded. Baseline characteristics of the 30 subjects who completed the study are shown in Table 1.

**Table 1:** Patient characteristics at baseline inclusion

Characteristics	Baseline *N* = 30 Median [IQR]
Age (years)	41.5 [34.0_51.3]
Gender, male no. (%)	15 (50)
BMI (kg/m^2^)	25.0 [22.5–28.3]
AHI (events/h)	2.5 [1.2–3.4]
% supine sleep of TST	40.3 [24.7–50.4]
Supine snore index	414.8 [252.8–699.5]
Non-supine snore index	205.9 [115.7–503.9]

*IQR* interquartile range, *BMI* body mass index, *TST* total sleep time, *AHI* apnea–hypopnea index

### SOS and SBPS scores

All individual scores are presented in Table 2. Complete sets of the SOS scores were collected in *n* = 19 study subjects. Another six subjects had missing data on one SOS item, for which we corrected. SOS data from a total of 25 subjects were available for analysis. SOS score in these subjects improved significantly from 35.0 ± 13.5 to 55.3 ± 18.6 (*p* < 0.001) after 6 weeks of SPT therapy. Total SBPS score (*n* = 24/30) also improved significantly from 24.7 ± 16.0 to 54.5 ± 25.2, *p* < 0.001.

**Table 2:** Individual results during baseline and at follow-up

Patient no.	Demographic characteristics			Polysomnographic parameters				Snoring questionnaires					
	Baseline			Baseline				Baseline			At 6 weeks		
	Gender	Age	BMI	AHI	% supine sleep	Supine SI	Non-supine SI	SOS	SBPS	VAS	SOS	SBPS	VAS
1	m	33	25.7	0.1	49.0	389.1	139.3	37.5	/	8	59.4	41.7	7
2	m	37	20.5	2.4	11.3	178.8	136.3	37.5	0.00	8	50.0	41.7	9
3	f	51	28.1	1.6	13.0	804.9	561.9	9.4	8.3	10	68.8	75.0	8
4	m	27	28.3	0.8	25.0	923	488.3	34.4	16.7	8	43.8	16.7	8
5	f	46	31.2	2.5	69.0	260.4	112.8	40.6	33.3	7	53.1	66.7	7
6	m	44	28.7	4.4	43.0	732.4	660	18.8	8.3	9	15.6	8.3	9
7	f	37	28.1	1.9	30.0	689.2	628.7	/	33.3	9	46.4	/	2
8	f	50	22.6	3.4	28.2	440.5	179.7	43.8	0.00	10	56.3	16.7	9
9	f	53	33.3	4.2	75.3	137.5	6.2	18.8	16.7	8	75.0	75.0	3
10	f	35	19.9	0.9	13.6	550.2	459	28.6	8.3	7	59.4	58.3	2
11	m	66	33.1	4.6	23.6	822.7	575.3	43.8	41.7	8	67.9	75.0	6
12	f	40	24.7	1.9	19.0	683.5	579.6	25.0	33.3	7	50.0	66.7	8
13	f	62	37.8	1.8	37.7	255.5	36	21.9	50.0	4	28.1	/	8
14	f	49	20.3	1.4	44.7	833.3	628.6	37.5	25.0	8	43.8	25.0	8
15	f	48	25.4	0.2	61.0	355.2	126.6	/	25.0	8	60.7	83.3	8
16	f	37	32.7	2.3	50.2	263.3	121.3	65.6	58.3	6	81.3	58.3	6
17	m	34	21.1	3.2	62.0	163.4	10	40.6	25.0	8	68.8	75.0	3
18	f	53	28.3	0.5	29.9	810.3	550.53	/	/	9	39.3	8.3	10
19	m	27	20.3	1	50.8	358.1	218.1	53.1	33.3	7	75.0	50.0	7
20	m	31	25	1.3	77.9	269.5	79	34.4	/	/	37.5	50.0	9
21	f	55	19.4	4.8	25.1	99.8	6.3	50.0	25.0	7	85.7	58.3	7
22	m	31	24.4	3	39.5	306.7	148.7	43.8	33.3	7	43.8	50.0	6
23	m	31	22.5	0.5	17.5	531.4	235.3	/	25.0	6	67.9	83.3	2
24	f	34	22.3	2.7	62.1	244.7	193.7	9.4	0.00	9	37.5	25.0	5
25	m	35	25	3.2	45.5	665.4	402.3	/	33.3	7	34.4	100.0	3
26	m	61	27.1	3.6	22.3	698.2	222	25.0	25.0	9	71.9	58.3	4
27	f	52	23.1	3.5	25.3	99.7	34.6	40.6	50.0	6	28.1	50.0	7
28	m	47	25	4.9	41.1	537.7	370.3	35.7	/	8	81.3	66.7	3
29	m	34	23.4	2.6	49.5	150.4	116.7	28.1	16.7	8	40.6	16.7	7
30	m	43	27.7	3	45.0	703.5	264.4	50.0	50.0	5	59.4	75.0	5
Mean ± SD								**35.0** ± **13.5**	**26.0** ± **16.2**	**7.6** ± **1.4**	**54.4** ± **17.8**	**52.7** ± **25.1**	**6.2 ± 2.4**

*BMI* body mass index, *AHI* apnea–hypopnea index, *SI* snoring index, *SOS* snore outcome survey, *SBPS* spouse/bed partner survey, *VAS* visual analogue scale, *f* female, *m* male, / missing value

### VAS score and satisfaction

Severity of snoring assessed by the bed partner (*n* = 29) decreased significantly from a median VAS score of 8.0 [7.0–8.5] to 7.0 [3.8–8.0] after 6 weeks (*p* = 0.004). Furthermore, 81.5% (*n* = 22/27) of the subjects reported that they were satisfied with the use of the SPT.

### Response rates

Twenty-four percent of the study subjects (*n* = 7/29) reported a VAS ≤ 3 after 6 weeks (‘major response’). A VAS ≤ 5 was seen in 34.5% (*n* = 10/29) and 36.0% (*n* = 9/25) had a SOS score ≥ 60.

A combination of VAS ≤ 5 and SOS score ≥ 60 was reported in four subjects 16.7% (‘fine response’).

## Discussion

This is the first prospective study investigating the effect of the SPT on position-dependent non-apneic snorers. After 6 weeks of therapy, we found a socially relevant improvement in the sleep-related health status of snorers and their bed partners, combined with high satisfaction rates assessed by the bed partners. VAS scores evaluating the severity of snoring on 10-point numeric scale did show a reduction as reported by the bed partners. However, since it reduced only by 1 point (8.0–7.0), we did not find a clinically relevant response. Although not all response rates were high, we found considerable improvements in the primary outcomes.

Non-apneic snoring is a prevalent problem with clinical and social implications. Since the literature suggests that a 68% of snorers are position dependent, new-generation PT could be very promising. There are several studies that have looked at the effect of PT in apneic snorers using old-generation positional devices: tennis ball techniques and pillows. Chen et al. studied whether a head-positioning pillow could reduce snoring sounds in patients with mild and moderate positional OSA. They found a significant reduction in VAS scale from 5.0 to 4.0 and snoring index from 218.0 events/h to 115.0 events/h [32]. These results are in line with the findings of our study where the VAS score also decreased with one point after therapy (from 8.0 to 7.0). Choi et al. studied the effect of PT using an inflatable vest-type device, in position-dependent snorers, with or without mild OSA [19]. A relevant effect was defined as a > 50% reduction of snoring rate in lateral position compared with the snoring rate in supine position. They found a significance decrease in snoring rate from 36.7 to 15.7%. Zuberi et al. also reported a significant reduction in snoring in patients with POSA treated with a triangular pillow [33]. In a study by Wenzel et al., patients with POSA were treated with a vest preventing the supine position [34]. A significance difference was found in snoring time (% of total sleep time) from 15.4 to 9.8%. However, there were other studies that did not find an improvement in snoring in apneic patients using PT [35–37]. We only found one study that evaluated the effect of PT in non-apneic snorers [38]. They used an anti-snoring pillow in primary snorers. Results reported that the snoring index significantly reduced from 269.0 to 162.5 and the mean snoring index was reduced by 39.6%. In the current study, only subjective parameters were evaluated and results showed that both SOS and SBPS scores improved significantly after 6 weeks.

There are not many other treatments available for positional or non-positional non-apneic snoring. Since snoring is regarded a social but non-medical condition, treatment is usually not reimbursed. Upper airway surgery for snoring can theoretically be applied, but often is overaggressive, irreversible, expensive, and not reimbursed [39]. Oral appliance therapy can be considered. However, it is often not reimbursed in non-apneic snoring. The effect of a cheaper option, the “boil and bite” oral device is often suboptimal, and not predictive of the effect of expensive custom-made titratable devices. Oral devices might have side effects such as painful jaws in the morning, dry mouth or hypersalivation, and long-term changes in occlusion. One-third of patients has a contra-indication for oral device therapy [40]. In case of insufficient effect or serious side effects, the considerable amount of money the patient has invested is lost. Continuous positive airway pressure (CPAP) can improve or eliminate snoring. Sériès et al. found that nasal CPAP (NCPAP) improves snoring in non-apneic snorers. In the NCPAP group, the snoring index decreased from 387/h to 320/h after therapy [41]. However, due to its low compliance, the limited acceptance, and high cost, CPAP is almost never used in the treatment of snoring [39]. Hence, PT in position-dependent non-apneic snoring may hold promising potential and results from the current study further highlight this potential. The SPT has been tested before in mild-to-moderate POSA patients by Van Maanen et al. and showed encouraging short and long-term results [26, 27]. These promising results in OSA patients were in line with other studies [28, 42]. The advantages of new-generation PT include that, in case it is not effective after a trial period, the device can be returned and side effects are limited.

Major limitations of our study are the uncontrolled design, the small cohort size, subjective outcome measures, and the short follow-up period. We report on short-term (6 weeks) effects, while a long-term effect is not investigated. However, from our experience with the SPT in OSA, we know that the long-term effect of the SPT remains stable [29]. Accurate measurement of objective snoring is difficult and for this study not possible. Furthermore, both a standardized definition of position-dependent snoring and strict and precise outcome measures to evaluate effect of anti-snoring treatment are lacking. The explanation for this is probably that non-apneic snoring is mostly a social problem, in contrast to OSA, which is a medical condition. This has made the necessity to determine objective snoring outcome measures less urgent and subjective outcomes more appropriate. Still, various parameters have been suggested in the literature to quantify snoring. But by far, the most relevant outcome in snoring is patient and bed partner satisfaction. To further define objective outcome measures and its effect on position-dependent non-apneic snorers, a large, prospective, longitudinal study is needed along with more long-term compliance data to truly assess the efficacy of PT in primary snoring.

## Conclusions

Due to the high prevalence of snoring in the general population and the associated negative mental and physical consequences, new therapeutic options are needed. The results of this study indicate that PT with the SPT improved several outcome measures in non-apneic position-dependent snorers. However, this non-controlled study has a short follow-up, so future studies are needed to review a controlled study design with longer follow-up period.

The results of this non-controlled study demonstrate that this SPT could potentially be considered as an alternative therapeutic option in the treatment regime of positional primary snorers.
